# Factors associated to bed net use in Cameroon: a retrospective study in Mfou health district in the Centre Region

**Published:** 2012-08-31

**Authors:** Viviane Hélène Matong Tchinda, Antoine Socpa, Aubin Armand Keundo, Francis Zeukeng, Clovis Tiogang Seumen, Rose Gana Fomban Leke, Roger Somo Moyou

**Affiliations:** 1 The Medical Research Centre, Institute of Medical Research and Medicinal Plant Studies (IMPM), Ministry of Scientific Research and Innovation, Yaoundé, Cameroon; 2 The Biotechnology Centre, University of Yaoundé I, Yaoundé Cameroon; 3 The Department of Anthropology, University of Yaoundé I, Yaoundé, Cameroon; 4 Ministry of Economy, Planning and Regional Development, Yaoundé Cameroon

**Keywords:** Malaria, factors, insecticides treated nets, health district, Cameroon

## Abstract

**Introduction:**

Insecticide treated net remains a tool of choice for malaria prevention in Cameroon. However, data suggests that its use by the population, especially vulnerable groups remains low. Moreover, there is a paucity of information about factors influencing its use. We sought out to identify factors associated with net use in Mfou health district, prior to distribution of long lasting insecticides treated nets (LLINs) in households.

**Methods:**

A two-stage cluster random sampling was conducted in 4 health areas with an average of 13 villages each. A total of 541 households were selected and heads interviewed using a structured household questionnaire. Data collected were entered into a database and multivariate logistic regression analyses of the association between net use and explanatory factors were performed using SPSS.

**Results:**

Net possession and use were respectively, 59.7 and 42.6%; thus, 2 out of 5 people who spent the previous night in households, slept under a net. Factors associated with net use included: net density≥0.5 (OR=8.88, 95% CI: 6.24-12.64), age≥5 years (OR=0.37, 95%CI: 0.28-0.47), secondary education (OR=1.41, 95% CI: 1.11-1.80) compared to primary/no education, parent status (OR=3.32, 95% CI: 2.31-4.76), house construction (OR=1.37, 95% CI: 1.10-1.71) and environment characteristics (OR=1.46, 95% CI: 1.18-1.80).

**Conclusion:**

These data suggest that a universal coverage with one LLIN for two people should be achieved in households. Then, malaria health education should be conducted to re-enforce net use among school-aged children and adolescents, as well as older household members. Moreover, management of environment and improvement in houses construction are necessary.

## Introduction

Malaria remains a major public health problem in Cameroon and control strategies adopted by the National Malaria Control Programme (NMCP) include: prompt management of confirmed cases with artemisinin-based combination therapy, promotion and distribution of insecticide-treated nets (ITNs), intermittent preventive treatment with sulphadoxine-pyrimethamine for pregnant women, environmental hygiene, etc. [1] Since adoption of ITNs as key preventive tool in 2002, several campaigns of free distribution of ordinary nets and latterly, long lasting insecticides treated nets (LLINs) were conducted all over the country, with priority given to pregnant women and children below five years. However, recent data [2] suggest that net possession and use remain low. While 52% of households possess any net, only 36% own an ITN, and only 28% of children below 5 years were reported to have slept under a net, with 21% under an ITN. One of the objectives of the Ministry of Health is to have 80% of children below five years sleeping under LLINs by 2015 [3]. Thus, to increase net possession, the NMCP has launched in August 2011 a national campaign for distribution of free LLINs to households, for universal coverage of the population. However, one of the greatest challenges is its effective use by individuals. Previous studies have shown that several factors influence net use. At individual level, these include: knowledge, beliefs, risks perception about malaria and perceived benefits of ITNs [4–12], age, sex and education level of individuals [10, 13–16]. Household level factors include: net density [10, 17–19], hanging status, paying for a net instead of obtaining free [20] household size, age, education and occupation of household head [10, 19–22], structure, space, types of sleeping units and intra-household sleeping arrangements [6, 7, 23] and household decision making processes [24]. In addition, net characteristics (age, shape, colour, condition, etc.) have also been associated with its use [10, 14, 19, 20, 25]. Other factors include climate and temperature associated with increased net use in the rainy season or reduced use during excessive heat [4, 26, 27], as well as socio-cultural and socio-economic activities which can temporary reduce net use even among regular net users [26–28]. However, many of these studies have analysed net use with respect to vulnerable groups (pregnant women and children below five years) while the current strategy is universal coverage of the whole population. In this study, we used an unbiased population to analyse and identify modifiable factors where efforts should be channelled to, in order to increase net use in households and further reduce malaria transmission.

## Methods

### Description of the study area

The study was conducted in Mfou health district in the Mefou and Afamba division, Centre region of Cameroon. This is a forest area, with a population estimated at about 71373 in 2011, mainly farmers and traders [29]. The headquarters of the district (Mfou urban) is a semi-urban area located at about 25 km from Yaoundé, the capital city of Cameroon. The climate is equatorial with four seasons, two wet and two dry. Several small streams from the Mefou and Afamba River irrigate major parts of the area. Mfou is hyperendemic for malaria and transmission occurs all year round, with peaks during the rainy season and transition to the dry season (March-June and September-October). A survey conducted in February 2011 showed that *Plasmodium falciparum* was the main malaria species, while *Anopheles gambiae* s.s. was the major malaria vector in the area (Tchinda et al., unpublished observations).

### Study design

This was a cross-sectional descriptive and analytical survey, conducted during the month of July 2011 after the rainy season. A two-stage cluster random sampling was used to select 4 health areas (Mfou, Nkilzok, Nkongoa and Nsimalen) among the 12 that constitute the district. The number of households per health area was selected proportionally to its population and sample size was calculated based on the proportion of children below 5 years who slept under any net the previous night [30].

A structured household questionnaire was used to capture individual characteristics (age, sex, education level, status in the household, main occupation, net use), household characteristics including: family size, living standard, net possession and their number, household head's knowledge about malaria, his perception of malaria as a serious disease, characteristics of house construction and the external environment surrounding households. Net characteristics (type, possession time, whether re-impregnated) were recorded as well. Interviews were conducted orally in French language and primarily with household heads or the spouses, and in case of absence, another adult member able to provide reliable information. Due to low level of education of some respondents in remote rural areas, community health workers provided translation to some of the questions when necessary in local language. The questionnaire was pre-tested in a non-survey area to determine the validity of pre-coded answers. An ethical clearance was obtained from the Institutional Ethics Committee of the Institute of Medical Research and Medicinal Plants Studies. Household heads gave their informed consent before initiation of the questionnaire.

### Data management and statistical analyses

Questionnaires were carefully reviewed after data collection, and data entry was performed in CSPro (version 4.0). All analyses (descriptive, univariate and multivariate) were performed in SPSS (version 18.0) and threshold for statistical significance was set at p<0.05. Analysis of the association between net use and independent variables was restricted to individuals living in households owning at least one net and who spent the previous night in households. The reported use/not use of a net the previous night was the dependent variable.

Net density was calculated by dividing the number of nets per household by the number of individuals and this was categorized as “low density” (ratio<0.5) and “high density” (ratio=0.5). The household living standard was derived from relevant characteristics that were discriminative in both rural and urban Mfou areas: “type of floor material”, “type of wall material”, “type of toilet”, and “presence of a clock”. This was done using multiple correspondence factor analysis. Households were categorized as having “low”, “medium” and “high living standard”. Household head's knowledge on malaria was based on the knowledge that mosquito bites transmit malaria and that sleeping under an ITN is currently the most effective method to prevent it. Respondents were categorized as having “good” or “average” knowledge. Perception of malaria as a serious disease was derived from three variables that determined whether malaria is: 1) a serious disease, 2) the leading cause of death in Cameroon and 3) the most prevalent disease in Cameroon. Respondents were categorized as having “good” or “average” perception about the seriousness of malaria. The environment of households was compounded from three variables including “presence of swampy areas/water ponds or rivers/streams”, “presence of bush/forest” and “household waste”. This was classified as environment “less suitable” or “very suitable” for mosquito proliferation. The characteristic of house was derived from three variables: “presence of a ceiling”, “presence of doors/or windows” and “presence of holes on walls”. This variable was classified as “secured” and “unsecured” for mosquito access.

All relevant variables that might explain the use of a mosquito net were used individually in univariate analysis (bivariate analysis of the association between each explanatory variable and the dependent variable). The relevance or choice of a variable was based on literature review (previous studies) and the context or environment of the study area. Only independent variables having a p-value≤0.10 in univariate logistic regression models were included in multivariate analysis. As no perfect or very high linear relationship should exist between two or more explanatory variables, a correlation matrix (multi-colinearity test) was performed with variables significant at this cut-off, in order to identify possible correlations. When two variables had a correlation coefficient of 0.6 or higher only one of them was included in the multivariate model. The correlation matrix showed that some variables such as age and occupation were highly correlated, which allowed us to eliminate the variable occupation. Variables were included one at a time, starting with the variable with greater statistical significance with the dependent variable. The variable not significant at p<0.05 was removed before inclusion of the subsequent variable, and so on and so forth, until the final model where all independent variables were significant was reached.

## Results

### Description of households, net possession and use rates

**Figure 1:**
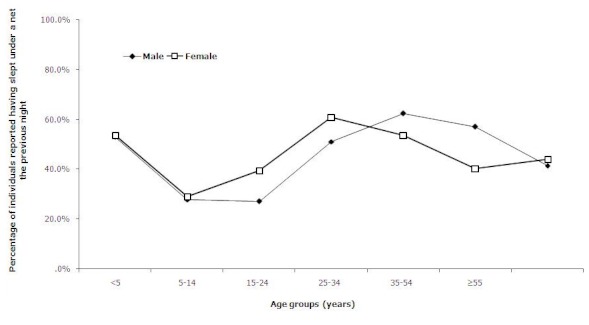
Age and sex-related use of net by individuals

A total of 323 households among the 541 surveyed, owned at least a net, giving a net possession rate of 59.7%, thus about 3 households out of 5. A total of 656 nets were declared in these households, with 18% being non-impregnated nets, 64.2% ordinary ITNs and 7% LLINs. The average number of nets per household was 2.0±1.2 with an average possession time of 2.3±1.6 years. Seventy nets (10.7%) were reported having been treated at least once with insecticides since an average period of 0.6 ± 0.7 year. In total, 4046 individuals were declared in households, 2259 among them resided in households owning at least a net and 962 slept under a net, giving a use rate of 42.6%. Table 1 gives the description of households, net possession and use rates. Looking at the age and sex-related use of net, the highest proportion of individuals using net the previous night was recorded in adults aged 25-49 years, followed by children aged below 5 years, while the lowest proportion was recorded among school-aged children and adolescents aged 5-24 years. No significant difference was observed with sex; however, females above 14 and less than 50 years tend to use nets more than men. Figure 1 shows the age and sex-related use of nets by individuals.

**Table 1:** Description of households, net possession and use rates

Characteristics	Area of residence		Total
	Urban Mfou	Rural Mfou	
Number of health areas	01	03	04
Number of households	231	310	541
Number of households owning at least a net	140	183	323
Proportion of households owing at least one net	60.6%	59.0%	59.7%
* Average number of nets own per household	2.1±1.2 (1-6)	1.9±1.2 (1-7)	2.0±1.2 (1-7)
Total number of individuals	1615	2432	4046
* Average family size per household	7.0±3.3 (1-22)	7.8±4.6 (1-29)	7.5±4.1(1-29)
Number of individuals in households owning at least a net	920	1339	2259
Proportion using net among the general population	43.2%	42.4%	42.6%
Proportion using net among children under 5 (N=637)	57,3%	50,5%	53.4%
Proportion using net among pregnant women (N=65)	44.4%	42.9%	43.6%

* Values are mean± standard deviation and range (minimum, maximum)

### Univariate logistic regression analysis of the association between net use and explanatory factors in households owning at least one net

At the individual level, factors significantly associated with net use were: children below five years (OR=1.66, 95% CI: 1.33-2.08) compared to individuals above five years; age 5-24 years (OR=0.39, 95% CI: 0.31-0.50) compared to children below five years; secondary education (OR=1.45, 95% CI: 1.18-1.78) and university level (OR=1.81, 95% CI: 1.26-2.61), compared to primary/no education. Regarding the main occupation of individuals (≥6years), unemployed persons or employed/retired ones had between 2-3 fold increases in net use compared to students (Table 2). Also, parents relative to their children in general, had close to a threefold increase in net use (OR=2.94, 95% CI: 2.39-3.63).

**Table 2:** Univariate analysis of factors associated with bed net use

Independent factors		Total persons N=2259	Number used net last night N=962	% used net last night	Odds ratio	95% CI*	p-value
Individual characteristics							
Age groups (years)	< 5	371	198	53.4%	1.0		
	5-24	1082	335	31.0%	0.39	0.31-0.50	0.000
	25-49	487	281	57.8%	1.20	0.91-1.57	0.194
	≥50	211	107	51.0%	0.91	0.65-1.27	0.57
Sex	Male	1030	425	41.4%	1.0		
	Female	1229	537	43.8%	1.10	0.93-1.30	0.24
Child < 5 years	No	1775	723	40.7%	1.0		
	Yes	371	198	53.4%	1.66	1.33-2.08	0.000
Pregnant woman	No	689	313	45.4%	1.0		
	Yes	39	17	43.6%	0.92	0.48-1.77	0.823
Education level	Primary/none	764	266	34.8%	1.0		
	Secondary	817	357	43.7%	1.45	1.18-1.78	0.000
	University	140	69	49.3%	1.81	1.26-2.61	0.001
** Main occupation	Students	949	285	30.0%	1.0		
	unemployed	286	134	46.9%	2.05	1.56-2.69	0.000
	Farmers	350	184	52.6%	2.58	2.00-3.32	0.000
	Employed	220	127	57.7%	3.18	2.35-4.30	0.000
Status in the household	Children	1409	525	37.4%	1.0		
	Parents	521	331	63.8%	2.94	2.39-3.63	0.000
	Other	320	105	32.8%	0.82	0.63-1.06	0.12
household size	Small	351	196	56.3%	1.0		
	Medium	702	349	49.9%	0.77	0.59-1.01	0.051
	Big	396	156	39.4%	0.50	0.38-0.67	0.000
	Very big	810	261	32.3%	0.37	0.29-0.48	0.000
Household living standard	Low	1140	479	42.2%	1.0		
	Medium	594	274	46.1%	1.17	0.96-1.43	0.122
	High	525	209	40.0%	0.91	0.74-1.12	0.382
Net density	Ratio < 0.5	1961	721	36.9%	1.0		
	Ratio≥0.5	298	241	80.9%	7.22	5.34-9.78	0.000
	Good	199	127	63.8%	1.24	0.75-2.06	0.402
House's construction	Secured	652	252	38.8%	1.0		
	Unsecured	1508	664	44.2%	1.25	1.04-1.51	0.019
Area of residence	Urban Mfou	920	397	43.2%	1.0		
	Rural Mfou	1339	565	42.4%	0.96	0.81-1.14	0.685
*** Characteristics of environment	Less suitable	1491	580	39.1%	1.0		
	Very suitable	703	350	49.9%	1.55	1.30-1.86	0.000

* CI: Confidence interval

** Analysis restricted to individuals aged≥6 years

*** Environment was characterized as been less or very suitable for mosquito proliferation.

At the household level, increasing net density was significantly associated with net use (OR=7.22, 95% CI: 5.34-9.78). While, increasing family size was significantly associated with a 50 and 63% reduction in net use by individuals, respectively in big (8-10 persons) and very big (≥11 persons) sized families, compared to small ones (≤5 people). Additionally, houses allowing easy access to mosquitoes (OR=1.25, 95% CI: 1.04-1.51) and environment favourable to mosquito proliferation (OR=1.55, 95% CI: 1.30-1.86) were all significantly associated with increased net use (Table 2). On the other hand, increased net use was observed among household heads with good knowledge on malaria (OR=1.38, 95% CI: 0.86-2.22) or good perception on its severity (OR=1.24, 95% CI: 0.75-2.06), compared to those with average or approximate knowledge or perception. However, no significant difference was observed between these two groups.

### Multivariate logistic regression analysis of the association between net use and explanatory factors in households owning at least one net

The final multivariate model consisted of 06 variables that were significantly associated with net use by individuals (Table 3). The most determinant factor associated with increasing odds of nets been used was its density in households. Individuals residing in households with one or more nets per two persons, had a 9 fold increase in chances of using a net, compared to those living in households with lower net density (OR=8.88, 95% CI: 6.24-12.64). Age was significantly associated with reduced net use, taking as reference group children aged less than five years. In fact, after adjusting with other factors, school-aged children and adolescents aged 5-24 years, adults aged 25-49 years and older household members (≥50 years) had respectively, 66, 41 and 71% reduction on chances of using net compared to this reference group. Also, parents (heads of households and their spouses) had a threefold increase in chances of using a net (OR=3.32, 95% CI: 2.31-4.76) compared to their children/grand children. In addition, household members with secondary education had 1.4 increase chances of using net (OR=1.41, 95% CI: 1.11-1.80) compared to individuals with primary/no education. Moreover, the external environment of households, favourable to mosquito proliferation (OR=1.46, 95% CI: 1.18-1.80) and houses with easy access to mosquito (OR=1.37, 95% CI: 1.10-1.71) remained significantly associated with net use in the final multivariate model.

**Table 3:** Multivariate logistic regression analysis of association between net use and explanatory factors in households owning at least one net

Independent factors		Odds ratio	95% CI	p-value	p-value test for trend
Net density in household	Ratio <0.5	1.0			
	Ratio≥0.5	8.88	6.24-12.64	0.000	
Age groups (years)	< 5	1.0			0.000
	5-24	0.34	0.26-0.44	0.000	
	25-49	0.59	0.41-0.85	0.005	
	≥50	0.29	0.18-0.48	0.000	
Child < 5 years	Yes	1.0			
	No	0.37	0.28-0.47	0.000	
Status in the household	Children	1.0			0.000
	Parents	3.32	2.31-4.76	0.000	
	Other	0.81	0.59-1.10	0.180	
* Education level	Primary or none	1.0			0.019
	Secondary	1.41	1.11-1.80	0.005	
	University	1.26	0.79-1.99	0.324	
Environment (suitable for mosquito proliferation)	Less suitable	1.0			
	Very suitable	1.46	1.18-1.80	0.000	
House construction characteristics	Secured	1.0			
	Not secured	1.37	1.10-1.71	0.005	

* The effect of education was assessed in 1822 individuals aged 6 years and above, after adjusting for others factors.

## Discussion

This study was designed to identify modifiable factors where efforts should be channelled to in order to accelerate effective net use in households of Mfou health district in Cameroon. Net use was expressed as the proportion of individuals in households owning at least one net, who slept under a net the previous night. Low net density was the most determinant factor of its use by individuals as previously observed [10, 17–19].

Children below five years were the group with high net use after adjusting with other factors, followed by adults aged 25-49 years, with a trend towards a high use in females, while school-aged children and adolescents and older household members remained the groups with lower net use. In fact, previous campaigns of distribution of free ITNs in Cameroon were given priority to pregnant women and children below five years. Moreover, mothers usually sleep with their nursing babies and young children, to make sure they are protected. Thus, the latter would be expected to use net more than other age groups. Conversely, older children usually sleep in separate bedrooms, with less attention to net use. This low net use in school-aged children and adolescents was previously observed [10, 13–15] and is a matter of concern, since the objective now is universal coverage of the population with LLINs, in order to achieve a significant reduction in malaria transmission. If the latter are not protected, they might significantly contribute to transmission when infected, as well as develop severe forms of malaria with increased mortality. Therefore, appropriate health education, targeting particularly this age group is necessary.

Additionally, net use seems to be a privilege for parents in the study locality of Mfou as previously observed.24 Parents here represent household heads and their spouses who have the decision making position in the household. In the context of scarcity of nets and high mosquito nuisance as it is the case in this forest area, bed nets are preferentially used to avoid nuisance and have a quiet night sleep as previously observed in studies conducted in rural African settings [5, 6, 11, 12, 24].

As previously reported [16, 21, 22], education of individuals was significantly associated with increased net use and this is related to the fact that through school education, people acquire biomedical knowledge on malaria, especially its transmission, consequences of the disease and preventive methods. Educated individuals are well prepared to integrate messages about the benefits of ITNs and adhere to its use.

Our findings also suggested an association between increasing net use and poor house's construction allowing easy access of mosquitoes, as well as the environment favourable to mosquito proliferation. In fact, Mfou health district is located in the midst of equatorial forest with many swampy areas, water ponds and rivers/streams, which constitute mosquitoes breeding sites. Moreover, most houses in the rural area are made up of mud and wood material, which sometimes have holes of walls. These houses often do not have a ceiling, or doors on bedrooms or windows. The abundance of mosquitoes creates a high nuisance, making populations to use mosquito nets. In line with these observations, previous studies have shown housing characteristics to be associated with increased mosquito numbers in homes [31].

Factors assessed in this study cannot explain in a holistic manner net use by individuals in households owning at least a net. Other factors could also contribute towards the understanding of this complex phenomenon. Moreover, qualitative studies are also necessary for the better understanding of individuals’ motivation in using net.

## Conclusion

Taken together, our data suggest that for an effective coverage of populations with LLINs, all household members should feel concerned with its use. Thus, while increasing net possession and density within households, it is of paramount importance to conduct appropriate health education targeting especially older children who might constitute reservoir for transmission if not protected. Also, efforts towards management of environment and improvement of house construction are needed to eliminate Anopheles breeding sites.

## References

[1] Ministry of Public Health, Cameroon/National Malaria Control Programme (2011). Report of activities of the NMCP for the year 2010 (in French).

[2] Cameroon DHS-MICS 2011 (2011). Preliminary report [PR13] (in French). http://www.measuredhs.com/pubs/pdf/PR13/PR13.pdf.

[3] Ministry of Public Health, Cameroon (2009). Health Sector Strategy 2001-2015.

[4] Binka N F, Adongo P (1997). Acceptability and use of insecticide impregnated bednets in northern Ghana. Trop Med Int Health..

[5] Adongo B Philip, Kirkwood Betty, Kendall Carl (2005). How local community knowledge about malaria affects insecticide-treated net use in Northern Ghana. Trop Med Int Health.

[6] Doannio CMJ, Doudou TD, Konan YL, Djouaka R, Toe PL, Baldet T, Akogbeto M, Monjour L (2006). Représentations sociales et pratiques liées à l'utilisation des moustiquaires dans la lutte contre le paludisme en Côte d'Ivoire (Afrique de l'Ouest). Med Trop..

[7] Léa Paré Toé, Olé Skovmand (2009). Decreased motivation in the use of insecticide-treated nets in a malaria endemic area in Burkina Faso. Malar J..

[8] Hwang Jimee, Graves M Patricia, Jima Daddi, Reithinger Richard, Kachur S Patrick, the Ethiopia MIS Working Group (2010). Knowledge of malaria and its association with malaria-related behaviours - results from the Malaria Indicator Survey, Ethiopia, 2007. Plos One.

[9] Dye DV Timothy, Apondi Rose, Lugada S Eric, Kahn G James, Smith Jacqueline, Othoro Caroline (2010). "Before we used to get sick all the time": perceptions of malaria and use of long-lasting insecticide-treated bed nets (LLINs) in a rural Kenyan community. Malar J.

[10] Graves M Patricia, Ngondi M Jeremiah, Hwang Jimee, Getachew Asefaw, Gebre Teshome, Mosher W Aryc, Patterson E Amy, Shargie B Estifanos, Zerihun Tadesse, Wolkon Adam, Reithinger Richard, Emerson M Paul, Richards O Frank (2010). Factors associated with mosquito net use by individuals in households owning nets in Ethiopia. Malar J..

[11] Louis PJ, Le Goff G, Trebucq A, Migliani R, Louis JF, Robert V, Carnavale P (1992). Feasibilité de la stratégie de lute par moustiquaires imprégnées d'insecticides rémanent en zone rurale au Cameroun. Ann Soc Belge Med Trop..

[12] Chambon R, Lemardelev P, Louis FJ, Foumane V, Louis JP (1997). Knowledge, attitudes and practice of populations faced with culicidae nuisances: results of 6 surveys taken in Cameroon in 1994. Bull Soc Pathol Exot..

[13] Noor M Abdisalan, Kirui C Viola, Brooker J Simon, Snow W Robert (2009). The use of insecticides treated nets by age: implications for universal coverage in Africa. BMC Pub Health.

[14] Tsuang Angela, Lines Jo, Hanson Kara (2010). Which family members use the best nets? An analysis of the condition of mosquito nets and their distribution within households in Tanzania. Malar J..

[15] Baume A Carol, Marin M Celeste (2007). Intra-household mosquito net use in Ethiopia, Ghana, Mali, Nigeria, Senegal, and Zambia: are nets being used? Who in the household uses them?. Am J Trop Med Hyg.

[16] Audrey Pettifor, Eboni Taylor (2008). Bed net ownership, use and perceptions among women seeking antenatal care in Kinshasa, Democratic Republic of the Congo (DRC): Opportunities for improved maternal and child health. BMC Public Health.

[17] Macintyre Kate, Keating Joseph, Okbaldt B Yohannes, Zerom Mehari, Sosler Stephen, Ghebremeskel Tewolde, Eisele P Thomas (2006). Rolling out insecticides treated nets in Eritrea: examining the determinants of possession and use in malarious zones during the rainy season. Trop Med Int Health.

[18] Eisele P Thomas, Keating Joseph, Littrell Megan, Larsen David, Macintyre Kate (2009). Assessment of insecticide-treated bednet use among children and pregnant women across 15 countries using standardized national surveys. Am J Trop Med Hyg.

[19] Carol A Baume, Ana Cláudia Franca-Koh (2011). Predictors of mosquito net use in Ghana. Malar J..

[20] Macyntire Kate, Littrell Megan, Keating Joseph, Hamainza Busiku, Miller John, Eisele P Thomas (2011). Determinants of hanging and use of ITNs in the context of near universal coverage in Zambia. Health Policy Plan.

[21] Ndjinga K Julie, Minakawa Noboru (2010). The importance of education to increase the use of bed nets in villages outside of Kinshasa, Democratic Republic of the Congo. Malar J..

[22] Alberto L García-Basteiro, Christopher Schwabe (2011). Determinants of bed net use in children under five and household bed net ownership on Bioko Island, Equatorial Guinea. Malar J..

[23] Iwashita Hanako, Dida Gabriel, Futami Kyoko, Sonye George, Kaneko Satoshi, Horio Masahiro, Kawada Hitoshi, Maekawa Yoshihide, Aoki Yoshiki, Minakawa Noboru (2010). Sleeping arrangement and house structure affect bed net use in villages along Lake Victoria. Malar J..

[24] Okrah Jane, Traoré Corneille, Palé Augustin, Sommerfeld Johannes, Muller Olaf (2002). Community factors associated with malaria prevention by mosquito nets: an exploratory study in rural Burkina Faso. Trop Med Int Health.

[25] Ngondi M Jeremiah, Graves M Patricia, Gebre Teshome, Mosher W Aryc, Shargie B Estifanos, Emerson M Paul, Richards O Frank, for the Ethiopia Malaria Indicator Survey Working Group (2011). Which nets are being used: factors associated with mosquito net use in Amhara, Oromia and Southern Nations, Nationalities and Peoples’ Regions of Ethiopia. Malar J..

[26] Pulford Justin, Hetzel W Manuel, Bryant Miranda, Siba M Peter, Mueller Ivo (2011). Reported reasons for not using a mosquito net when one is available: a review of the published literature. Malar J..

[27] Alaii JA, Hawley WA, Kolczak MS (2003). Factors affecting use of permethrin-treated bed nets during a Randomized controlled trial in western Kenya. Am J Trop Med Hyg..

[28] Dunn E Christine, Le Mare Ann, Makungu Christina (2011). Malaria risk behaviours, socio-cultural practices and rural livelihoods in southern Tanzania: implications for bednet usage. Soc Sci Med..

[29] Equipe Cadre du District Santé de Mfou (2011). Plan d'action opérationnel du district pour l'année 2011.

[30] MICS3 Cameroon. Final report (in French) (2006). http://www.childinfo.org/files/MICS3_Cameroon_FinalReport_2006_Fr.pdf.

[31] Kirby J Matthew, Green Clare, Milligan M Paul, Sismanidis Charalambos, Jasseh Momadou, Conway J David, Lindsay W Steven (2008). Risk factors for house entry by malaria vectors in rural town and satellite villages in Gambia. Malar J..

